# Clinical and Subclinical Congestion in Acute Heart Failure: A Multimodal Prognostic Assessment

**DOI:** 10.3390/jcm15072601

**Published:** 2026-03-29

**Authors:** Sara Lozano-Jiménez, Paula Vela-Martín, Alba Martín-Centellas, Daniel de Castro, Cristina Mitroi, Francisco José Hernández-Pérez, Marta Cobo-Marcos, Sergio Martínez-Álvarez, Manuel Gómez-Bueno, Javier Segovia-Cubero, Jesús Álvarez-García, Mercedes Rivas-Lasarte

**Affiliations:** 1 Department of Cardiology, Hospital Universitario Clínico de Salamanca, 37007 Salamanca, Spain; sara12s@hotmail.com; 2 Department of Cardiology, Hospital Universitario de Lleida, 25198 Lleida, Spain; 3 Department of Cardiology, Instituto de Investigación Sanitaria Puerta de Hierro Majadahonda (IDIPHISA), Hospital Universitario Puerta de Hierro Majadahonda, 28222 Madrid, Spainsergiomaral27@gmail.com (S.M.-Á.); jsecu@telefonica.net (J.S.-C.); 4 CIBER Cardiovascular, Instituto de Salud Carlos III, 28029 Madrid, Spain; 5 Department of Cardiology, Hospital Universitario Ramón y Cajal, 28034 Madrid, Spain

**Keywords:** venous excess ultrasound score, congestion, subclinical congestion, lung ultrasound, heart failure

## Abstract

**Background/Objectives:** Congestion is a hallmark of heart failure (HF) and a major determinant of outcomes. Non-invasive tools enable detection of subclinical congestion, but their correlation and prognostic relevance remain incompletely defined. The present study aimed to assess the prevalence, evolution, interrelationships, and prognostic impact of clinical and subclinical congestion markers in patients hospitalized for HF. **Methods**: This single-centre, prospective cohort study included adults admitted with HF who underwent serial evaluations at admission, 72 h, pre-discharge, early outpatient follow-up and at 6 months. Clinical congestion was assessed using a standardized physical examination score. Subclinical congestion was evaluated using lung ultrasound (LUS), Venous Excess Ultrasound Score (VExUS), and Remote Dielectric Sensing (ReDS). Patients were classified according to the presence of clinical and/or subclinical congestion at discharge. The primary endpoint was a composite of all-cause mortality, HF readmission, or unscheduled visits requiring intravenous diuretics within six months. **Results**: Ninety-four patients (mean age 74 ± 11 years, 68% male) were included. While clinical congestion improved significantly during hospitalization, approximately 30% of patients remained clinically congested at discharge. Among clinically euvolemic patients, only 47% showed no evidence of subclinical congestion. Correlations between congestion markers were weak to moderate, suggesting complementary pathophysiological information. At discharge, pulmonary B-lines were the strongest predictor of the composite endpoint (hazard ratio [HR] 3.50, 95% CI 1.41–8.72), followed by clinical congestion (HR 2.67, 95% CI 1.13–6.30). Patients with clinical and subclinical congestion exhibited lower event-free survival. **Conclusions**: Subclinical congestion is common despite apparent clinical euvolemia and is associated with worse outcomes. Integrating clinical assessment with non-invasive congestion markers may improve post-discharge risk stratification in HF.

## 1. Introduction

Heart failure (HF) represents a significant and growing public health challenge, with a rapidly increasing prevalence [1]. It remains one of the leading causes of hospitalization and mortality worldwide. A central pathophysiological feature of HF is the development of systemic and pulmonary congestion, primarily driven by elevated intracardiac filling pressures and complex alterations in fluid distribution and venous capacitance. Beyond simple fluid accumulation, congestion reflects a dynamic interplay between cardiac dysfunction, neurohormonal activation, renal impairment, and increased venous pressures, ultimately leading to organ dysfunction and adverse clinical outcomes. Clinically, congestion manifests through symptoms and physical signs such as peripheral oedema, pulmonary rales, and jugular venous distension. It is a hallmark of HF and a well-established predictor of hospitalization, disease progression, and mortality [2]. However, clinical assessment alone has limited sensitivity and may fail to detect early or residual congestion [3,4,5]. Increasing evidence indicates that subclinical congestion frequently persists despite apparent clinical euvolaemia, particularly during the vulnerable post-discharge period, and may contribute to early rehospitalization and poor prognosis [6].

Importantly, increased intracardiac filling pressures frequently precede the development of overt congestive symptoms by days or even weeks [4]. Consequently, the early detection of congestion before clinical decompensation occurs is of utmost importance. However, in its initial stages, subclinical congestion frequently remains undetected by routine clinical assessments, including physical examination (PE) or chest radiography.

In recent years, several novel non-invasive methods have been developed to detect subclinical congestion, increasingly recognized as a key precursor of worsening HF and hospitalization. Among these, lung ultrasound (LUS) has emerged as a particularly robust tool with substantial evidence to assess pulmonary congestion through the detection of more than three B-lines, which reflect increased extravascular lung water [7,8,9]. Additionally, the Venous Excess Ultrasound Score (VExUS) has been proposed as a comprehensive assessment of systemic venous congestion, integrating evaluation of the inferior vena cava (IVC), suprahepatic, portal, and renal veins [10]. Other promising tools include radar-based technology such as the Remote Dielectric Sensing (ReDS) system, which allows the quantification of lung fluid content using electromagnetic signals and has been validated as a tool to estimate pulmonary congestion [11]. Circulating biomarkers, including natriuretic peptides, further complement these approaches by reflecting cardiac wall stress and volume overload [12].

However, most previous studies have evaluated individual markers of congestion in isolation, often focusing on either pulmonary or systemic congestion. Data integrating multiple congestion domains within the same cohort, particularly during the vulnerable transition from hospitalization to early post-discharge follow-up, remain limited.

Therefore, this study aimed to provide a comprehensive, multimodal assessment of congestion, integrating clinical, pulmonary, and systemic markers, and to evaluate their temporal evolution and prognostic relevance in patients hospitalized for HF.

## 2. Methods

### 2.1. Study Design and Population

This single-centre, prospective cohort study was conducted between June 2021 and March 2023 at a tertiary hospital. All consecutive adults admitted to the cardiology ward with a primary diagnosis of HF, defined according to current European HF guidelines [13], were considered eligible for inclusion. Patients on dialysis, presenting cardiogenic shock at admission, or with an estimated life expectancy of less than six months due to non-cardiovascular causes were excluded. Patient screening and inclusion were dependent on operator availability, and no formal screening log of all eligible patients was maintained.

### 2.2. Study Protocol

Participants underwent five predefined assessments: (1) at hospital admission (within 24 h), (2) at 72 h, (3) prior to discharge, (4) at an outpatient HF clinic visit 1–2 weeks after discharge, and (5) a 6-month follow-up telephone call to collect events. Each visit included a standardized clinical evaluation, measurement of NT-proBNP, lung ultrasound (LUS), VExUS, and ReDS. Jugular venous pressure and hepatojugular reflux were assessed according to standard clinical practice with the patient in a semi-recumbent position (30–45°). Given the pragmatic design of the study, physical examination was performed by experienced heart failure cardiologists without a strictly standardized protocol, reflecting real-world clinical assessment. Patient management was left to the discretion of the treating physicians and was not guided by protocol. Additional details regarding the study protocol are provided in the Supplementary Materials.

Briefly, LUS was performed by assessing eight lung zones (four in each hemithorax), with the transducer oriented perpendicular to the ribs and the patient in a semi-recumbent position. ReDS was obtained using the Pro System version of the device. Values equal or less than 35% were considered normal, whereas values above 35% were considered indicative of pulmonary congestion. The VExUS score evaluated four parameters: IVC size, hepatic vein Doppler waveform, portal vein pulsatility, and renal vein flow pattern. Congestion severity was categorized as mild (one abnormal parameter), moderate (two abnormal parameters), or severe (three abnormal parameters).

Thresholds were selected based on previously published literature evaluating pulmonary and systemic congestion in heart failure. Lung ultrasound B-lines have been widely validated as markers of pulmonary congestion [14], while ReDS values > 35% have been associated with increased lung fluid content [11]. VExUS ≥ 1 was used to identify early systemic venous congestion [15]. In addition, sensitivity analyses were performed using a stricter threshold for systemic congestion (VExUS ≥ 2).

Based on discharge findings, patients were categorized into three groups according to the presence or absence of clinical and subclinical congestion. Subclinical congestion was defined by any of the following criteria: more than two lung zones with more than 3 B-lines, ReDS > 35% or VExUS ≥ 2. Clinical congestion was defined as the presence of rales, jugular venous distension or peripheral oedema.

### 2.3. Study Outcomes

The primary outcome was a composite endpoint of all-cause mortality, HF readmissions (>24 h), or unscheduled visits due to HF requiring intravenous diuretics at a 6-month follow-up.

### 2.4. Statistical Analysis

Continuous variables were expressed as mean ± standard deviation or median with interquartile range and compared using ANOVA or Wilcoxon tests. Categorical variables were presented as counts and percentages, with χ^2^ or Fisher’s tests. Correlation analyses were performed to explore the relationships between different clinical and subclinical congestion markers, with the aim of assessing whether these techniques provide overlapping or complementary information across different congestion domains. Time-to-event analyses utilized Kaplan–Meier curves, and Cox proportional hazards regression identified predictors of the primary outcome. Follow-up was administratively censored at 6 months. Patients lost to follow-up were censored at the time of last contact. Statistical significance was set at a *p* value < 0.05. Analyses were conducted using STATA, version 18 (Stata Corp., College Station, TX, USA).

Given the exploratory nature of this prospective cohort study and the limited number of outcome events, the primary analyses were performed using univariable Cox proportional hazards models to avoid model overfitting. Model discrimination was evaluated using Harrell’s C-statistic using bootstrap resampling with 1000 replications to derive 95% confidence intervals. Because of the small number of deaths observed during follow-up, competing risk analyses were not performed.

## 3. Results

### 3.1. Clinical Characteristics of the Study Population

A total of 94 patients (68% males, mean age of 74 ± 11 years) were included in the study, with 5 in-hospital deaths. Patients presented with cardiovascular risk factors and a high burden of comorbidities. Most patients were in NYHA functional class II or III. The most frequent HF aetiologies were valvular heart disease and ischemic cardiomyopathy.

The mean left ventricular ejection fraction (LVEF) was 46 ± 15%. Baseline clinical characteristics, including metabolic parameters, electrolyte levels, and guideline-directed medical therapy, are summarized in Table 1.

### 3.2. Trajectories of Clinical and Subclinical Congestion

The clinical congestion score decreased significantly from 4.0 ± 2.1 at admission to 0.3 ± 0.6 at discharge (*p* < 0.001). However, approximately 30% of patients remained clinically congested at discharge. Regarding subclinical congestion, around 33% of patients remained congested according to LUS, 30% according to VExUS, and up to 22% according to ReDS. When considering these three complementary diagnostic methods, only 47% of patients who were clinically deemed euvolemic at discharge showed no signs of subclinical congestion. While both clinical and subclinical congestion parameters improved during the hospital stay, they worsened at the first outpatient visit. The temporal evolution of congestion is detailed in Table 2.

### 3.3. Correlation Between Clinical and Subclinical Congestion

The correlation between congestion parameters is shown in Table 3. Pulmonary B-lines showed a moderate correlation with crackles (0.54) and weakly with ReDS (0.38), while ReDS correlated minimally with other measures. Right-sided congestion assessed by VExUS showed moderate correlations with hepatomegaly (0.40), peripheral oedema (0.31), hepatojugular reflux (0.29), and the clinical congestion score (0.38).

### 3.4. Prognostic Impact

**Figure 1:**
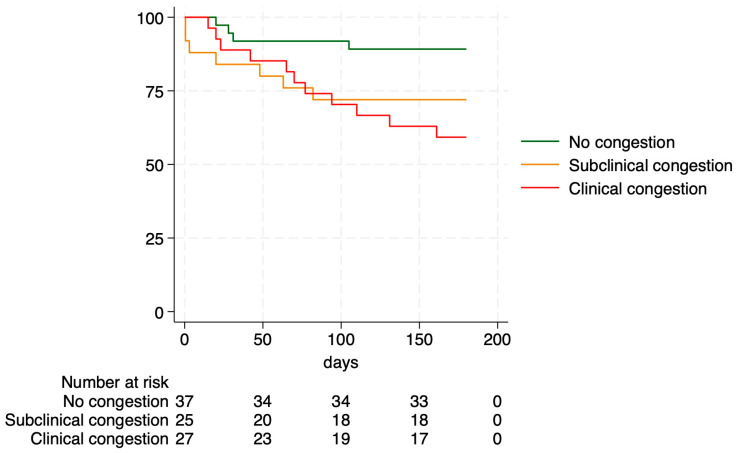
Kaplan–Meier curves according to the presence of clinical or subclinical congestion at discharge for the combined endpoint in a 6-month follow-up.

The combined endpoint occurred in 25 patients (26.9%), with some patients experiencing more than one event. In univariate Cox regression models (Table 4), the presence of B-lines was associated with the six-month composite endpoint, with a hazard ratio (HR) of 3.5 (95% confidence interval [CI]: 1.41–8.72), followed by clinical congestion at discharge (HR 2.67; 95% CI: 1.13–6.30), portal vein pulsatility (HR 1.92; 95% CI: 1.01–3.66), and IVC diameter (HR 1.07; 95% CI: 1.01–1.13). Harrell’s C-indices are reported in the Supplementary Materials. Kaplan–Meier analysis showed a trend towards worse outcomes in patients with subclinical congestion [HR 3.00 (0.88–10.3)], although this did not reach statistical significance, and significantly worse outcomes in patients with clinical congestion [HR of 4.22 (1.34–13.26)], Figure 1.

## 4. Discussion

This study provides novel insights into the prevalence and clinical significance of subclinical congestion at hospital discharge in patients with acute HF. Although most patients were assessed as euvolemic on physical examination, a substantial proportion remained congested when evaluated with other tools such as LUS, VExUS, or ReDS. Notably, less than half of the clinically euvolemic patients were free of any subclinical congestion, highlighting the limitations of relying solely on conventional bedside assessment. Interestingly, markers of clinical and subclinical congestion exhibited only weak-to-moderate correlations, supporting a complementary role of these parameters in assessing different facets of congestion and supporting the concept that congestion is not uniformly distributed.

### 4.1. Prognostic Relevance of Congestion

Congestion continues to be a major determinant of outcomes in HF, contributing to both readmissions and mortality. However, it is well established that bedside signs of congestion have limited sensitivity and are subject to interobserver variability when compared with invasive hemodynamic assessment [3,5], which supports the complementary use of non-invasive imaging tools. A key strength of this study was the integrated evaluation of congestion across multiple compartments, combining clinical assessment with pulmonary and systemic venous markers within the same cohort. This approach allowed a more comprehensive characterization of congestion, which is often heterogeneous and incompletely captured by a single modality. In this cohort, the six-month combined event rate was substantial, reflecting the persistent vulnerability of this population. Importantly, both subclinical and clinical congestion at discharge were associated with a worse prognosis at six months, although the association with subclinical congestion did not reach statistical significance, likely due to the limited number of events and wide confidence intervals.

### 4.2. Prognostic Value of Pulmonary Ultrasound (B-Lines)

One of the most consistent findings in our cohort was the association between B-lines at discharge and adverse outcomes. Patients displaying two or more scan fields with three or more B-lines had an approximately threefold higher risk of the composite endpoint at six months. In line with these findings, B-lines showed the highest discriminative performance among the evaluated markers, although overall discrimination was modest and confidence intervals overlapped across variables. Therefore, these results should be interpreted with caution and do not imply superiority over other congestion markers.

These results are consistent with previous studies and meta-analyses demonstrating that higher B-line burden is associated with worse outcomes, with reported AUC values approaching 0.8 when ≥5 B-lines are detected. Furthermore, ultrasound-guided diuretic strategies have been associated with improved clinical outcomes, including reductions in composite endpoints of 45–49% and number needed to treat as low as 5–8 patients [7,16]. However, most previous studies have evaluated pulmonary congestion in isolation, whereas the present study integrates pulmonary and systemic markers within the same cohort.

### 4.3. Prognostic Value of ReDS

ReDS also identified residual pulmonary congestion in a substantial proportion of patients, although its prognostic value was less pronounced than B-lines. Previous studies have evaluated the use of ReDS to guide therapy. Alvarez-García et al. demonstrated that ReDS-guided management significantly reduced adverse events (2% vs. 20%) using a discharge threshold of <35% [11]. Although statistical significance was not reached in our cohort, we observed a mean reduction in ReDS values at discharge, which is clinically relevant and consistent with prior studies.

### 4.4. Prognostic Value of Systemic Venous Congestion (VExUS)

VExUS has emerged as a useful tool for the assessment of systemic venous congestion. Initially developed in cardiac surgery patients [17], its ability to reflect elevated filling pressures, renal dysfunction, and diuretic response has subsequently been supported by several studies in patients with decompensated HF [18,19,20]. In addition, prior studies have associated higher VExUS grades with an increased risk of in-hospital mortality, as well as higher rates of readmission and death within 90 to 180 days after discharge [21].

Although VExUS ≥ 1 did not reach statistical significance for the composite endpoint, it showed moderate correlations with clinical signs such as oedema, hepatomegaly, and hepatojugular reflux. A trend towards a higher risk of adverse events was observed, suggesting that systemic venous congestion may still provide incremental prognostic information, particularly through parameters such as IVC diameter and portal vein pulsatility.

Correlations among congestion parameters were mostly weak to moderate supporting the concept that congestion is a multi-compartmental process, with different techniques capturing distinct physiological aspects rather than interchangeable measurements. Lung ultrasound appears to align more closely with clinical pulmonary findings, whereas VExUS may better reflect systemic venous congestion. In contrast, ReDS showed limited concordance with other parameters in this cohort. From a practical perspective, if simplification of congestion assessment is considered in routine clinical practice, combining focused lung ultrasound with selected systemic venous markers (such as IVC diameter and portal vein pulsatility) may provide a pragmatic and clinically informative approach.

### 4.5. Limitations

Several limitations of this study should be acknowledged. First, the lack of blinding may have introduced bias, potentially limiting the generalizability of the findings. In addition, patient inclusion was partly dependent on operator availability, and no formal screening log of all eligible patients was maintained, which may have introduced selection bias.

Furthermore, the choice of the primary endpoint was influenced by both methodological and organisational factors. On the one hand, the relatively small sample size, limited number of events, and six-month follow-up constrained the statistical power and precluded more complex multivariable modelling. On the other hand, the structure of the HF unit, which provides rapid access to unscheduled visits, may have influenced event rates and reflects local care pathways.

In this context, the exploratory nature of the study should be acknowledged, as it was likely powered to detect larger prognostic effects, while smaller associations may have gone undetected. Additionally, competing risk analyses were not performed due to the low number of deaths observed during follow-up.

Finally, practical considerations such as cost, accessibility, and operator dependency may limit the widespread implementation of the proposed multimodal assessment. The absence of offline echocardiographic review and interobserver variability analyses also represents a limitation. Therefore, the present findings should be interpreted with caution and considered hypothesis-generating.

In addition to imaging-based congestion assessment, several other tools are increasingly available to assist clinicians in HF management, including biomarkers, implantable hemodynamic monitoring systems, and digital health technologies. These strategies may provide complementary information for the early detection of worsening heart failure and are increasingly being incorporated into contemporary heart failure care pathways [22].

## 5. Conclusions

Most patients who appeared clinically euvolemic exhibited residual congestion. The presence of both clinical and subclinical congestion was associated with lower event-free survival at a six-month follow-up. These findings suggest that integrating non-invasive tools may improve post-discharge risk stratification. Larger prospective studies are warranted to validate these observations and establish their role in routine clinical practice.

## Figures and Tables

**Table 1:** Baseline characteristics of the study population.

Variable	N (%)
**Sex, male**	64 (68.0)
BMI	28 (26–31)
**Comorbidities:**	
Hypertension	65 (69.1)
Diabetes mellitus	35 (37.2)
Dyslipidaemia	49 (52.1)
History of smoking	39 (41.5)
History of alcoholism	18 (19.1)
Atrial fibrillation	
None	25 (26.7)
Paroxysmal	21 (22.3)
Persistent	13 (13.8)
Permanent	35 (37.2)
Chronic kidney disease (glomerular filtration < 60 mL/min/m^2^)	37 (39.4)
Chronic obstructive pulmonary disease	16 (17.0)
Sleep apnoea-hypopnea syndrome	10 (10.9)
Stroke	9 (9.9)
Peripheral vascular disease	5 (5.4)
**HF profile:**	
First episode of HF	8 (8.5)
NYHA functional class	
I	2 (2.1)
II	58 (61.7)
III	31 (33.0)
IV	3 (3.2)
Aetiology	
Valvular	25 (26.7)
Ischemic	20 (21.6)
Idiopathic	15 (16.0)
Hypertensive	14 (14.9)
Restrictive	10 (10.9)
Other	9 (9.9)
LVEF category	
Preserved (≥50%)	55 (58.5)
Intermediate (41–49%)	20 (21.3)
Reduced (<40%)	19 (20.2)
**Laboratory data at discharge**	
Leukocyte count (cells per microliter)	6920 (5710–8680)
Glycosylated haemoglobin	5.8 (5.6–6.4)
Sodium (mmol/L)	139 (137–141)
Potassium (mmol/L)	4.2 (3.8–4.4)
Creatinine (mg/dL)	1.3 (1.0–1.7)
Ferritin (ng/mL)	114 (49–208)
Transferrin saturation (%)	13 (9–19)
**Echocardiography data**	
LVEF (%)	45 (35–59)
Left atrial size, volume (mL)	49 (39–67)
TAPSE (mm)	17 (15–20)
PAPS (mmHg)	40 (25–50)
**Treatment at discharge**	
ACEi/ARBs	7 (7.4)
Sacubitril/valsartan	12 (12.8)
Beta-blockers	57 (60.6)
MRA	14 (14.9)
SGLT2i	20 (21.3)
Furosemide < 120 mg	35 (37.2)
Furosemide ≥ 120 mg	59 (62.8)
Thiazide	14 (14.9)
Digoxin	2 (2.1)

Abbreviations: NYHA: New York Heart Association, HF: heart failure; LVEF: left ventricular ejection fraction, TAPSE: Tricuspid annular plane systolic excursion, PAPS: estimated pulmonary artery systolic pressure, ACEi: Angiotensin-Converting Enzyme inhibitors, ARB: Angiotensin Receptor Blockers, MRA: Mineralocorticoid Receptor Antagonists, SGLT2i: Sodium-Glucose Transport 2 Inhibitors. Results are expressed as a number (percentage) or median (interquartile range).

**Table 2:** Evolution of clinical and subclinical congestion through HF admission.

Variable	Admission (n = 94)	72 h (n = 93) *	Pre-Discharge (n = 89) *	HF Clinic Visit After Discharge (n = 79)	*p* Value
**Orthopnoea**					<0.001
None	28 (29.8)	67 (72.0)	86 (96.6)	68 (90.6)	
Sometimes	14 (14.9)	22 (23.6)	3 (3.4)	4 (5.5)	
Always	18 (19.1)	1 (1.1)	0	1 (1.3)	
Paroxysmal nocturnal dyspnoea	34 (36.2)	3 (3.3)	0	2 (2.6)	
**Crackles**					<0.001
None	20 (21.3)	69 (74.2)	86 (96.6)	67 (89.3)	
Basal	64 (68.1)	23 (24.7)	3 (3.4)	8 (10.7)	
Mid	10 (10.6)	1 (1.1)	0	0	
**Pleural effusion**	26 (27.6)	8 (8.6)	3 (3.4)	6 (8.1)	<0.001
**Weight, kg**	75.8 ± 18.1	73.5 ± 16.9	71.7 ± 17.2	72.6 ± 15.6	<0.001
**Oedema**					<0.001
None	18 (19.1)	54 (58.1)	77 (86.5)	54 (72.0)	
Malleolar	36 (38.3)	27 (29.0)	12 (13.5)	16 (21.3)	
Knee	17 (18.1)	8 (8.6)	0	3 (4.0)	
Above knee	23 (24.5)	4 (4.3)	0	2 (2.7)	
**Hepatomegaly**					<0.001
None	46 (48.9)	69 (74.2)	81 (91.0)	52 (69.6)	
1–2 fingerbreadths	28 (29.8)	21 (22.6)	7 (7.9)	19 (24.0)	
>2 fingerbreadths	20 (21.3)	3 (3.2)	1 (1.1)	4 (6.4)	
**Jugular venous distension**					<0.001
None	39 (41.5)	62 (66.7)	72 (80.9)	47 (62.7)	
<SCM	33 (35.1)	27 (29.0)	17 (19.1)	24 (32.0)	
SCM	14 (14.9)	3 (3.2)	0	3 (4.0)	
Mandible	8 (8.5)	1 (1.1)	0	1 (1.3)	
**Hepatojugular reflux**	37 (39.4)	19 (20.7)	7 (7.9)	14 (18.9)	<0.001
**Clinical congestion score**	4.0 ± 2.1	1.5 ± 1.6	0.3 ± 0.6	0.8 ± 1.3	<0.001
NT-ProBNP, pg/mL	6180 (3749–11,162)	3406 (1655–7085)	2713 (1046–5423)	3414 (818–6642)	0.003
CA125, U/mL	37.5 (21–62)	48 (12–86)	40 (11–40)	Not available	<0.001
Creatinine, mg/dL	1.4 (0.7)	1.5 (0.8)	1.4 (0.7)	1.6 (0.7)	0.472
Presence of B-lines, n (%)	97%	82%	52%	54%	<0.001
No. of zones with ≥3 B-lines	4.8 ± 2.0	2.7 ± 2.1	1.3 ± 1.8	1.8 ± 2.2	<0.001
ReDS, %	34.0 ± 7.6	32.3 ± 6.1	29.9 ± 6.5	29.2 ± 5.9	<0.001
IVC diameter, mm	22.6 ± 5.1	19.7 ± 4.9	18.2 ± 5.3	18.7 ± 6.1	<0.001
Suprahepatic veins					<0.001
S > D	28 (33.7)	37 (43.5)	41 (51.2)	35 (54.7)	
D > S	21 (25.3)	20 (23.5)	13 (16.2)	6 (25.0)	
Inversion	34 (41.0)	28 (33.0)	26 (32.6)	23 (20.3)	
Portal vein pulsatility					<0.001
<30%	31 (39.7)	47 (58.8)	53 (61.7)	35 (54.7)	
30–50%	29 (37.2)	22 (27.5)	16 (25.0)	13 (20.3)	
>50%	18 (23.1)	11 (13.7)	13 (20.3)	8 (10.1)	
Renal Doppler	<0.001				
Continuous	26 (32.1)	43 (50.6)	48 (59.3)	30 (46.9)	
Biphasic	41 (50.6)	37 (42.0)	26 (32.1)	22 (34.4)	
Monophasic	14 (17.3)	6 (7.4)	7 (8.6)	12 (18.7)	
VExUS score, mean ± SD	1.3 ± 1.0	0.95 ± 1.0	0.64 ± 1.0	0.97 ± 1.3	<0.001
VExUS points, n (%)					
0	22 (25.6)	45 (51.1)	60 (69.8)	39 (57.4)	
1	30 (34.9)	21 (23.9)	8 (9.3)	9 (13.2)	
2	20 (23.2)	14 (15.9)	11 (12.8)	5 (7.4)	
3	14 (16.3)	8 (9.1)	7 (8.1)	15 (22.0)	

* N = 93 at 72 h due to death of 1 patient; n = 89 at discharge due to death of 5. SCM: Sternocleidomastoid muscle. *p* value for the overall comparison among the four columns; CA125: Cancer Antigen 125. D: Diastolic. HF: Heart Failure. IVC: Inferior Vena Cava. Mg/dL: Milligrams per decilitre. mm = Millimetres. NT-proBNP: N-terminal pro–B-type Natriuretic Peptide. Pg/mL: Picograms per millilitre. ReDS: Remote Dielectric Sensing. SD: Standard deviation. S: Systolic. U/mL: Units per millilitre. VExUS: Venous Excess Ultrasound Score.

**Table 3:** Correlation between clinical and subclinical congestion markers.

Variable	VExUS	B-Lines	NT-ProBNP	Creatinine	ReDS	Congestion	Crackles	Hepatomegaly	HJR
**B-lines**	0.36 *								
**NT-proBNP**	0.26 *	0.29 *							
**Creatinine**	0.11 *	−0.02	0.25 *						
**ReDS**	0.23 *	0.38 *	0.09	0.11					
**Congestion**	0.38 *	0.53 *	0.30 *	0.02	0.23 *				
**Crackles**	0.17 *	0.54 *	0.18 *	−0.03	0.26 *	0.57 *			
**Hep**	0.40 *	0.38 *	0.29 *	0.10	0.03	0.63 *	0.25 *		
**HJR**	0.29 *	0.21 *	0.19 *	0.09	0.08	0.53 *	0.23 *	0.51 *	
**Oedema**	0.31 *	0.41 *	0.21 *	−0.03	0.11	0.83 *	0.46 *	0.53 *	0.41 *

* Statistically significant. Congestion: clinical congestion. ReDS: Remote Dielectric Sensing system. HJR: hepatojugular reflux. White: no correlation. The code of colors yellow ⟶ green ⟶ orange ⟶ red indicates the degree of correlation from weaker to stronger correlations.

**Table 4:** Cox regression model for the composite endpoint.

Variable	HR	95% CI	*p* Value
Presence of B-lines *	3.50	1.41–8.72	0.007
NT-ProBNP	1.00	0.99–1.00	0.09
ReDS > 35%	2.05	0.69–6.13	0.20
Clinical congestion	2.67	1.13–6.30	0.02
VExUS ≥ 1	1.84	0.73–4.68	0.20
VExUS ≥ 2	1.93	0.72–5.15	0.19
IVC diameter	1.07	1.01–1.13	0.01
Portal vein pulsatility	1.92	1.01–3.66	0.05
SH	1.38	0.82–2.32	0.22
Renal Doppler	1.44	0.77–2.68	0.25
Subclinical pulmonary congestion **	2.73	1.10–6.79	0.03

HR: Hazard ratio; CI: Confidence interval; ReDS: Remote Dielectric Sensing; SH: Suprahepatic veins; IVC: Inferior vena cava; VExUS: Venous Excess Ultrasound. * Two or more zones with three or more B-lines. ** Two or more zones with three or more B-lines or ReDS > 35%.

## Data Availability

The data presented in this study are available on request from the corresponding authors.

## Supplementary Materials

The following supporting information can be downloaded at: https://www.mdpi.com/article/10.3390/jcm15072601/s1, Study protocol for the assessment of subclinical congestion.
