# Supplementary material for: Clinical and Subclinical Congestion in Acute Heart Failure: A Multimodal Prognostic Assessment
Source: J Clin Med. 2026 Mar 29;15(7):2601. doi: 10.3390/jcm15072601 (PMC13073270; doi:10.3390/jcm15072601)
Supplement: Supplementary file 1 [file jcm-15-02601-s001.zip › jcm-4190395-supplementary.pdf]

Supplementary material:

Study protocol for the assessment of subclinical congestion.

LUS: Lung ultrasounds were performed using a Philips CX50 ultrasound system. An 8-quadrant protocol was applied, with four quadrants on each hemithorax, following current recommendations. A cardiac probe was used, and examinations were conducted with the patient in supine position. All images were recorded and stored for later review. B-lines were defined as ≥3 vertical artifacts per field arising from the pleural line, with a "comet-tail" appearance, hyperechogenicity, and synchronous movement with pleural sliding.

ReDS: For the evaluation of pulmonary congestion with ReDS®, the patient's anthropometric data—weight, height, and sex—were first entered into the device. Next, the thoracic circumference was measured with a tape measure and entered in centimeters. The vest size was then adjusted according to the device's instructions (A, B, C, or D). Finally, the device was placed on the right hemithorax with the patient seated, ensuring proper support of the back against a firm surface to prevent movement. After a 45-second measurement, the device provided the result as a percentage of lung water. Each patient's measurement was recorded for subsequent review.

VExUS: Venous Excess Ultrasound Score involves:

· The diameter of inferior vena cava (IVC) which is measured two centimeters from its entrance into the right atrium in the subcostal plane. In the presence of volume overload, dilation (greater than 21 mm) is observed, accompanied by a collapse of less than 50% during deep inspiration.

· Pulsed doppler flow in:

o Portal vein: under physiological conditions, the pulsatility index is typically less than 30%. As systemic congestion intensifies, a notable increase in pulsatility becomes evident, exceeding 50% in cases of marked impairment.

o Renal veins (arcuate and interlobar vessels): in euvolemic individuals, a continuous waveform is obtained. In mild congestion, a biphasic discontinuous pattern may be documented. Subsequently, as venous overload progresses, a monophasic discontinuous signal may emerge.

o Hepatic veins: systolic phase is characterized by greater amplitude compared to the diastolic component (S>D). In slight congestion, the pattern shifts to D>S. As fluid retention worsens, a reversed systolic wave might be identified.

Variable	Harrell's C-index (95%CI)
Presence of B-lines*	0.651 (0.537-0.763)
NT-ProBNP	0.640 (0.416-0.769)
ReDS >35%	0.569 (0.503-0.696)
Clinical congestion	0.610 (0.516-0.729)
VExUS ≥1	0.566 (0.499-0.685)
VExUS≥2	0.560 (0.501-0.669)
IVC diameter	0.619 (0.500-0.766)
Portal vein pulsatility	0.622 (0.513-0.769)
SH	0.571 (0.500-0.699)
Renal Doppler	0.606 (0.486-0.713)
Subclinical pulmonary congestion**	0.624 (0.516-0.737)
